# Glucoraphanin and sulforaphane biosynthesis by melatonin mediating nitric oxide in hairy roots of broccoli (*Brassica oleracea* L. var. *italica* Planch): insights from transcriptome data

**DOI:** 10.1186/s12870-022-03747-x

**Published:** 2022-08-17

**Authors:** Shaoying Ma, Jinyu Bao, Yaqi Lu, Xu Lu, Peng Tian, Xiaoling Zhang, Jie Yang, Xiaotong Shi, Zhihui Pu, Sheng Li

**Affiliations:** 1 Basical Experimental Teaching Center, Gansu Agricultural University, Lanzhou, 730070 Gansu Province China; 2 College of Horticulture, Gansu Agricultural University, Lanzhou, 730070 Gansu Province China; 3 College of Life Science and Technology, Gansu Agricultural University, Lanzhou, 730070 Gansu Province China

**Keywords:** Glucoraphanin, Sulforaphane, Broccoli hairy roots, Nitric oxide, Hydrogen peroxide, Transcriptomics

## Abstract

Glucoraphanin (GRA) is present in the seeds and nutrient organs of broccoli and is the precursor of the anti-cancer compound sulforaphane (SF). The hairy roots obtained by infecting broccoli (*Brassica oleracea* L. var. *Italic* Planch) leaves with *Agrobacterium rhizogenes* (ATCC15834) are phytohormonally autonomous, genetically stable, and can produce large amounts of the anti-cancer substance SF. Melatonin (MT) is a natural hormone widely found in plants. Studies have shown that melatonin can regulate the synthesis of secondary metabolites of downstream targets by mediating the synthesis of signal molecules. However, whether MT regulates the synthesis of NO and H_2_O_2_ and mediates the synthesis mechanism of secondary metabolites, GRA and SF, is not yet clear. In this study, the hairy roots of broccoli were treated with 500 μmol/L MT, and the genome of broccoli (*Brassica oleracea* L. var. *botrytis* L) was used as the reference genome for transcriptome analysis. By this approach, we found that MT regulates the synthesis of NO and H_2_O_2_ and mediates the synthesis of secondary metabolites GRA and SF. GO annotations indicated that DEGs involved in the MT treatment of broccoli hairy roots were mainly related to catalytic activity, cells, and metabolic processes; the KEGG pathway analysis indicated that MT treatment likely affects the hormone signal transduction process in broccoli hairy roots; broccoli hairy roots were treated with 500 μmol/L MT for 0, 6, 12, 20, and 32 h, respectively; compared with 0 h, the yield of GRA and SF increased under the other treatments. The highest yields of GRA and SF occurred at 12 h. The NO content was the highest at 12 h, and the H_2_O_2_ content was positively correlated with MT concentration. The content of NO and H_2_O_2_ were regulated, and the content of GRA and SF was increased under MT treatment. NO synthase inhibitor (L-NAME and TUN) could effectively inhibit the content of NO in broccoli hairy roots and reduce GRA and SF yield; MT could regulate NO levels by regulating NO synthesis-related enzymes and could alleviate the reduction of NO content in tissue cells caused by NO synthase inhibitor and promote NO synthesis. These results have important theoretical implications for understanding the regulation of GRA and SF synthesis events by NO and H_2_O_2_.

**Supplementary Information:**

The online version contains supplementary material available at 10.1186/s12870-022-03747-x.

## Introduction

Glucosinolates (GLS) are natural products rich in sulfur anions. GLS is divided into aliphatic GLS, aromatic GLS, and indole family Indolyl [1]. GLS is chemically stable and widely found in cruciferous plants. The GRA in aliphatic GLS is the precursor of the active substance sulforaphane (SF) [2]. When plant tissues are damaged, GRA is released from the plant vacuole and is quickly hydrolyzed upon contact with myrosinase (MYR) to form a molecule of glucose and an unstable aglycone. Then, depending on the chemical environment, the aglycone intermediates spontaneously rearrange into compounds, such as isothiocyanates (ITC), thiocyanates, nitriles, and oxazolidine-2-thione; among them, the production of isothiocyanates under neutral conditions has attracted attention [3].

SF is one of the isothiocyanate. SF has a strong pharmacological activity, especially with preventive and anti-cancer effects. SF is one of the substances with the strongest anti-cancer activity [4]. Studies have shown that SF can inhibit the activation of protocarcinogens substances and promote the decomposition of carcinogenic chemicals in the body, thereby achieving cancer prevention effect. In addition, SF also has anti-oxidation [5] and anti-inflammatory [6] functions. Therefore, the study of SF activity has become a research hotspot.

In recent years, broccoli has attracted much attention because of its richness in GRA and SF, but SF is mainly obtained from broccoli seeds or seedlings, which have problems such as low yield and high production cost. Studies have shown that the ability of the hairy roots of *Forsythia* to synthesize secondary metabolites is improved relative to *Forsythia* [7], indicating that the hairy roots culture technique can solve the problem of insufficient SF synthesis. Our laboratory successfully established a broccoli hairy roots culture system [8]. It was found that the SF content in broccoli hairy roots was 13.07- and 31.28-fold higher than that in the leaves and roots of sterile broccoli seedlings, respectively, as detected by HPLC [9]. For SF production, broccoli hairy roots have an advantage over other Brassica plants.

MT is a pleiotropic molecule involved in various physiological processes in plants. It has been reported that MT can be used as an antioxidant to remove reactive oxygen species (ROS) and other harmful oxidative molecules in plant cells [10]. At present, some researchers have reported that MT can promote the synthesis of secondary metabolites in plants. Zhang et al. [11] found that 100 μmol/L MT pretreatment up-regulated anthocyanin biosynthesis genes in kale at the transcriptional level and promoted the accumulation of kale anthocyanins. Wei et al. [12] found that 100 μmol/L MT treatment significantly increased the expression of GLS biosynthesis-related genes in broccoli, and the contents of total GLS and SF in broccoli were significantly increased. However, there are no reports about MT regulating GRA synthesis in broccoli hairy roots and its transformation to SF.

Certain concentrations of MT increase NOS-dependent NO production and decrease mitochondrial membrane potential [13]. In the normal physiological process of plants, oxygen metabolism is in a dynamic equilibrium state, but ROS is produced when plants are subjected to external stress [14]. Many studies have demonstrated that ROS is not only a product of plant oxygen metabolism but can also be used as a second messenger to participate in the signal transduction process of cells [15]. So far, the main NO synthesis pathways identified are the NOS (nitric oxide synthase, NOS) and NR (nitrate reductase, NR) pathways. The NOS pathway is mainly dependent on NO synthase (NOS) [16], which generates NO by oxidizing L-arginine [17], but the activity of this protein can be reduced by N-nitro-L-arginine methyl (L-NAME), which is an inhibitor of NOS in animal cells. NO can also be produced by peroxidase (POD), where NO is generated from N-hydroxy-L-arginine (NOHA) and H_2_O_2_ catalyzed by POD; polyamine oxidase (PAO) can also be converted from polyamines (PA) to produce NO, and NADH/NADPH can be oxidized by cytochrome oxidase/reductase to produce NO. For the NR pathway, nitrate reductase (NR) can reduce NO_3_^−^ to NO_2_^−^, and then nitrite reductase (NiR) can reduce NO_2_^−^ to NO, and NR can also oxidize NO to nitrate to scavenge NO, which shows that NR is an important regulator of NO homeostasis in plants [18], and xanthine oxidase from buttermilk (XOR) and nitric oxide reductase (Ni-NOR) are also involved, and the NR pathway can be mediated by inhibition of tungstate (TUN).

Plants produce NO [19], and in addition to the above-mentioned NO synthesis pathways, there are also some non-enzymatic NO synthesis pathways, such as glutathione (GSH) or ascorbic acid (ASA). NO can also be produced in an acidic environment [20]. NO is a gas signal molecule. The role of NO has become clearer as scholars have studied it intensively. Many studies have proved that NO is involved in the growth and development of plants. NO not only regulates the formation of lateral roots and root hairs but also mediates adventitious root formation during growth hormone (Indole-3-acetic acid, IAA)-induced plant physiology [21]. NO also plays an important role in regulating the synthesis of secondary metabolites. Zhao et al. [22] reported that exogenous NO acts as a signaling molecule involved in the biosynthesis and regulation of perylenequinone in bamboo.

There are many synthesis pathways for H_2_O_2_ in plant cells, such as H_2_O_2_ production via the plasma membrane redox system (PRMS) [23] and H_2_O_2_ synthesis via POD [24]. The functions of H_2_O_2_ in plants are also diverse. The oxidation capacity reduces peroxidative damage in plants [25]. In terms of regulating the synthesis of secondary metabolites, Jahan et al. [26] have reported that H_2_O_2_ is involved in regulating the synthesis of plant secondary metabolites, and Sharma et al. [27] show that H_2_O_2_ can interact with other signal molecules, especially hormone signals, to mediate plant signal transduction. At low concentrations, ROS can regulate the biosynthesis of plant secondary metabolites. Wu et al. [28] report that H_2_O_2_ is involved in the signal transduction process of MeJA-induced ginsenoside synthesis in the ginseng adventitious roots culture system. NO is one of the important signaling molecules in plants. It participates in various signal transduction processes in plants and is of great significance for maintaining normal growth, development, and physiological homeostasis in plants.

In this study, broccoli hairy roots were treated with 500 μmol/L MT for 0, 6, 12, 20, and 32 h. The broccoli (*Brassica oleracea* L. var. *italica* Planch) genome was used as the reference genome, and RNA-seq sequencing technology was applied to investigate the effects of MT-mediated NO and H_2_O_2_ on GRA and SF synthesis in broccoli hairy roots. The present study focused on verifying the mechanism of NO-mediated MT regulating GRA and SF biosynthesis in broccoli hairy roots. This study’s findings provide a new perspective for exploring the complex molecular mechanism of MT’s regulation of GRA and SF by mediating NO in hairy roots of broccoli.

## Results

### Effect of MT treatment for different times on the yields of GRA and SF in broccoli hairy roots culture system

**Fig. 1:**
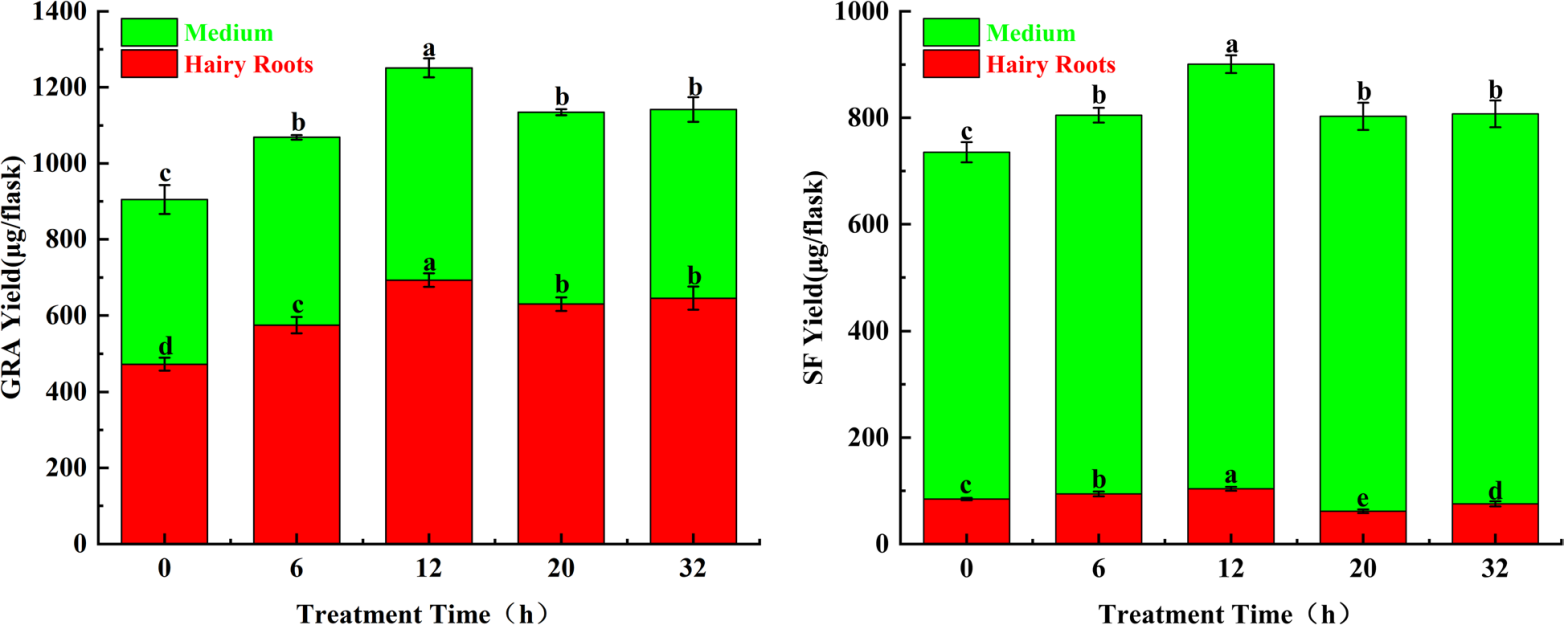
The GRA and SF contents in the hairy roots culture system. Values followed by different lowercase letters represent *P* ˂ 0.05

MT treatment significantly changed the yields of GRA and SF in the broccoli hairy roots culture system. The yields of GRA and SF were the highest at 12 h, and the yields of all treatments were higher than the control (0 h). For SF, the yield of all treatments in the medium was higher than that in hairy roots. However, the yield of GRA in the hairy was higher than medium (Fig. 1).

### Effect of MT treatment for different times on the content of NO and H_2_O_2_ in broccoli hairy roots

**Fig. 2:**
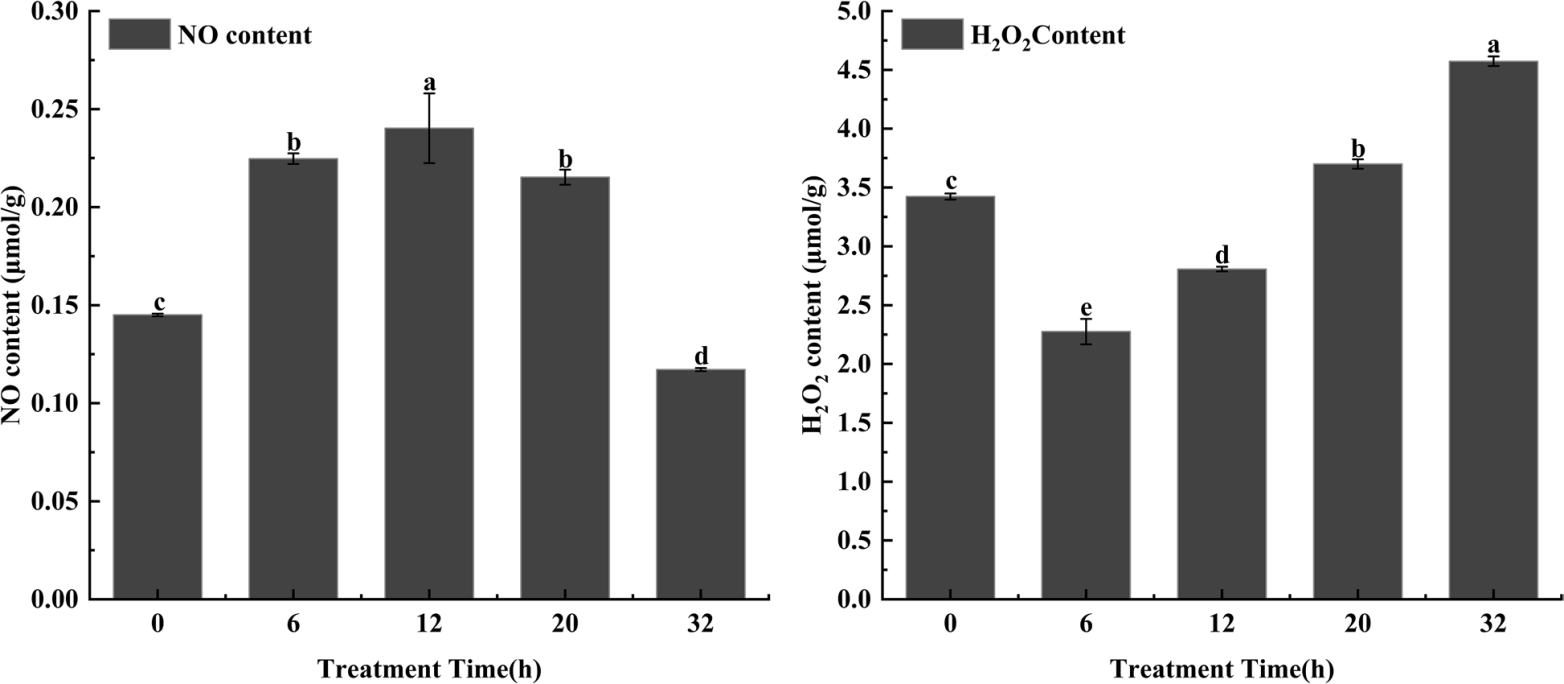
The effect of MT treatment for a different time on the content of NO and H_2_O_2_ in broccoli hairy roots. Values followed by different lowercase letters represent *P* ˂ 0.05

Broccoli hairy roots grown for 20 days were treated with 500 μmol/L MT for 0, 6, 12, 20 and 32 h. The content of NO and H_2_O_2_ in hairy roots was detected by the kit. The NO content was the highest at 12 h, and this was significantly different from the other treatments (*P*˂0.05). H_2_O_2_ content was the highest at 32 h and was significantly different from the control and other treatments (Fig. 2).

### Transcriptome sequencing and annotation

Using the hairy roots of broccoli from the 0.5 mmol/L MT treatment, an average of 24.71 Gb clean data were generated from the 15 samples, with Q30 values > 93.90% (Table S1). The unigenes annotation information was obtained by comparing the GO and KEGG databases. The original transcriptome data of this manuscript and article https://doi.org/10.1080/15592324.2021.1952742 are consistent, but these articles have distinct focuses.

### Analysis of differentially expressed genes (DEGs)

**Fig. 3:**
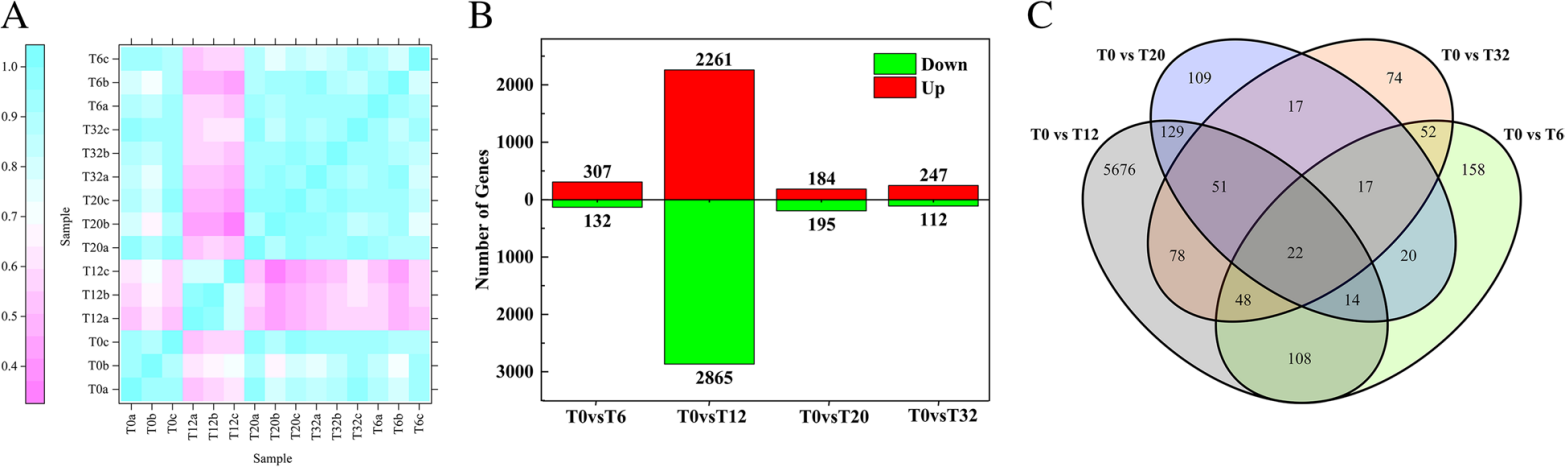
(**A**) Heatmap of the Pearson’s correlation between samples. The x- and y-axes represent each sample. (**B**) A column diagram represents the numbers of up-regulated and down-regulated DEGs in two groups. (**C**) Venn diagrams represent the numbers of DEGs and the overlaps of sets obtained across four comparisons

The expression levels of the unigenes were presented as FPKM values. The correlations were calculated between every two samples, and they showed high intra-group correlations (Fig. 3A), which indicated good sample consistency. In the hairy roots of broccoli from the 0.5 mmol/L MT treatment, a total of 439, 5126, 379, and 359 DEGs were found in hairy roots broccoli subjected to T0 vs. T6, T0 vs. T12, T0 vs. T20, and T0 vs. T32, respectively (Fig. 3B). Twenty-two DEGs were found in hairy roots of broccoli at all four time points (T0 vs. T6, T12, T20, and T32) (Fig. 3C).

### Functional classification and enrichment analysis of DEGs

**Fig. 4:**
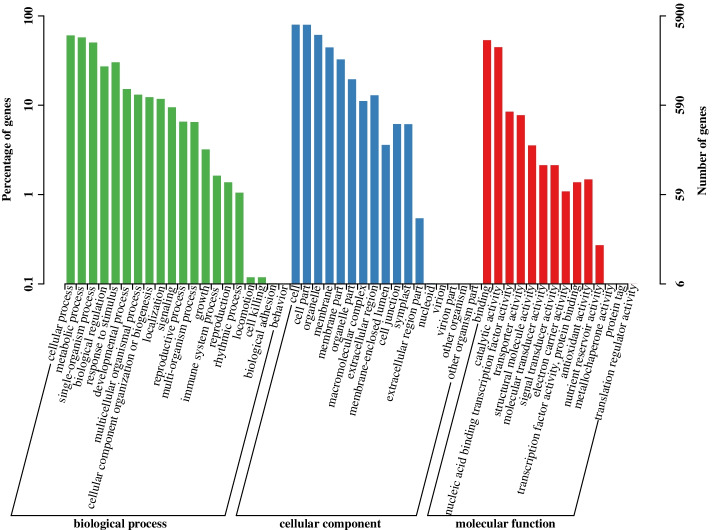
GO analysis of DEGs for T0 vs. T6, T0 vs. T12, T0 vs. T20 and T0 vs. T32 in three main categories. The x-axis represents GO terms belonging to three categories; the y-axis represents the gene numbers and gene proportion for each term

The functional classification of identified DEGs under MT treatment in hairy roots of broccoli was performed using the GO annotation system. A total of 5900 DEGs in T0 vs. T6, T0 vs. T12, T0 vs. T20, and T0 vs. T32 were annotated and divided into 41 level-2 functional classifications consisting of 11 terms of molecular function, 12 terms of cellular component, and 18 terms of biological process. The top three terms of molecular function were binding (GO: 0,005,488), catalytic activity (GO: 0,003,824), and nucleic acid binding transcription factor activity (GO: 0,001,071). Most of the terms in the cellular component were annotated in cell (GO: 0,005,623), cell part (GO: 0,044,464), and organelle (GO: 0,043,226). The terms of the biological process are mainly related to the cellular process (GO: 0,009,987), metabolic process (GO: 0,008,152), and single-organism process (GO: 0,044,699) (Fig. 4). These results indicated that the DEGs involved in MT treatment hairy roots of broccoli were mainly related to catalytic activity, cell, and metabolic process.

### KEGG pathway and enrichment analysis of DEGs

**Fig. 5:**
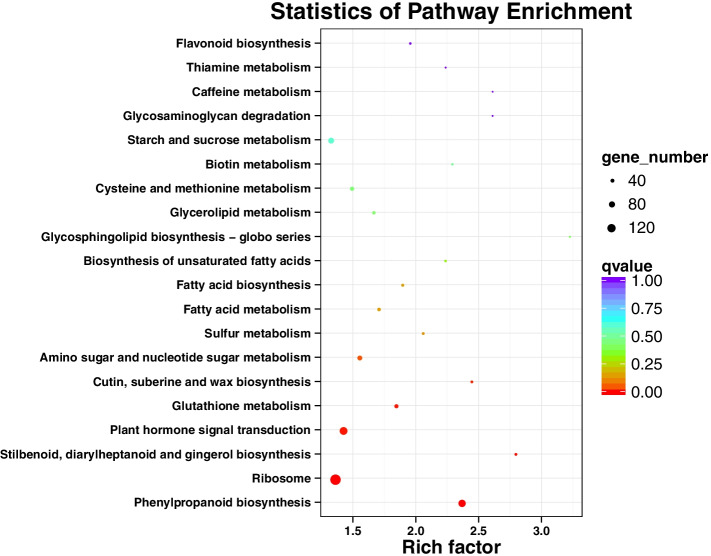
KEGG pathway enrichment of the DEGs. The y-axis represents KO terms; the x-axis represents the rich factor

Our KEGG pathway enrichment analysis showed that the DEGs were significantly enriched in phenylpropanoid biosynthesis (ko: 00,940), ribosome (ko: 03,010), stilbenoid, diarylheptanoid and gingerol biosynthesis (ko: 00,945), plant hormone signal transduction (ko: 04,075), and glutathione metabolism (ko: 00,480) (Fig. 5).

### Expression analysis of genes related to NO synthesis in hairy roots of broccoli under MT treatment

**Fig. 6:**
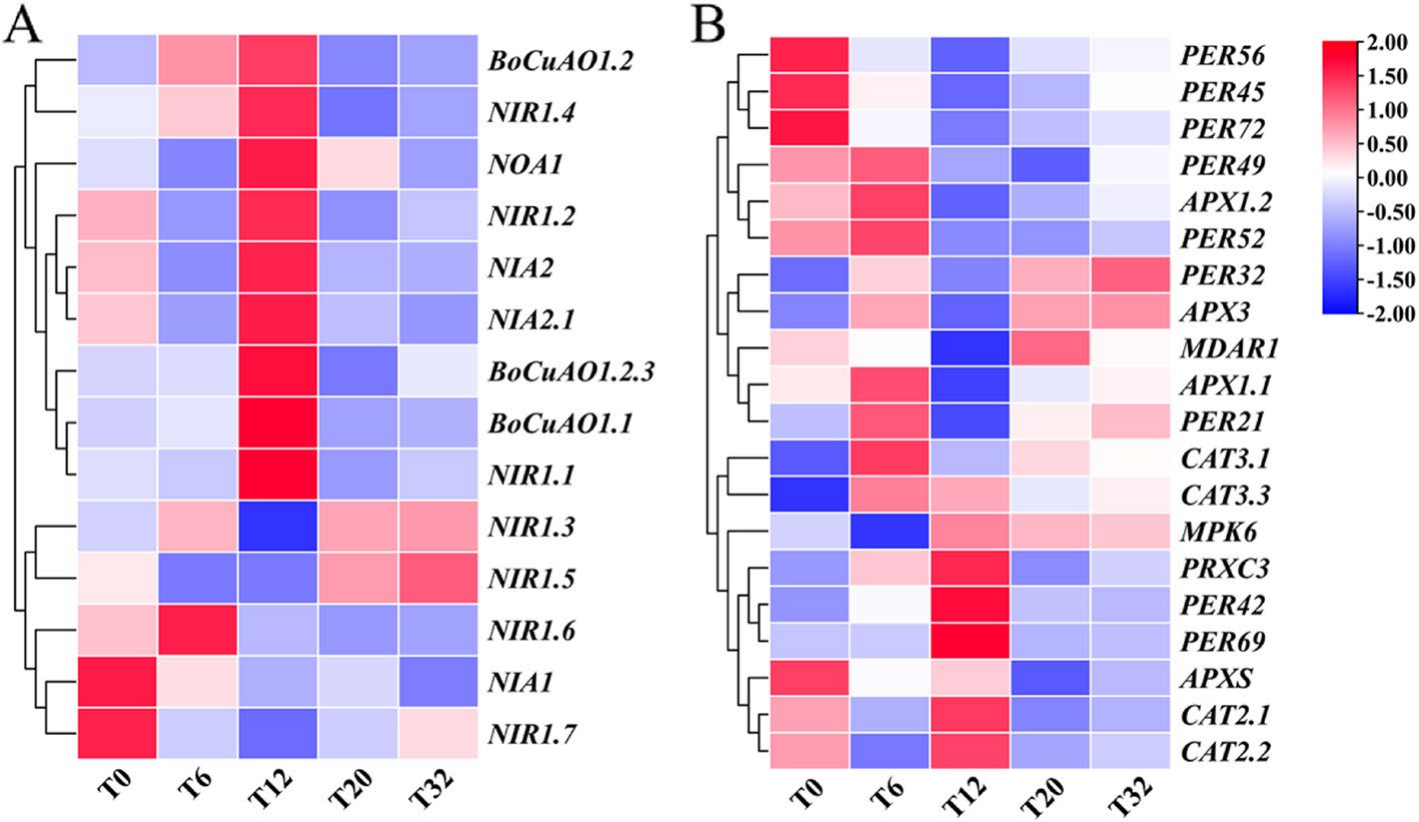
Cluster analysis of NO (**A**) and H_2_O_2_ (**B**) synthesis genes in hairy roots of broccoli

The addition of MT not only induced the synthesis of GRA and SF, the secondary metabolites of broccoli hairy roots but also affected the production of the signaling molecule NO. The plant signal molecule NO plays a key role in many physiological processes, such as plant growth and development. The experiment screened the differentially expressed genes related to NO metabolism from the transcription level of broccoli hairy roots (Fig. 6A). These genes encode multiple enzymes involved in NO synthesis, such as nitric oxide synthase (*NOA1*), nitrate reductase (*NIA1/NIA2*), nitrite reductase (*NIR1*), and copper amine oxidase family (*BoCuAO1*). Compared with MT treatment of broccoli hairy roots at 0 h, *BoCuAO1.1*, *NIR1.4*, *NOA1*, *NIA2*, *NIR1.2*, *BoCuAO1.2.3*, *BoCuAO1.2*, and *NIR1.1* genes were up-regulated at 12 h. At the same treatment time (12 h), *NIR1.3*, *NIR1.5*, *NIR1.6*, *NIR1.7*, and NIA1 expressions were down-regulated. At 6 h, *NIR1.6* was highly expressed. The overall expression of NO metabolism-related genes was low when treated with MT for 20 h and 32 h.

### Expression analysis of genes related to H_2_O_2_ synthesis in hairy roots of broccoli under MT treatment

Using broccoli hairy roots, we detected differentially expressed genes (at the transcriptome level) related to antioxidants (Fig. 6B). Among them were peroxidase-encoding genes, *CAT* family genes, *PER* family genes encoding the synthesis of peroxidase, and *APX* family genes encoding ascorbateperoxidase. Compared with MT treatment, *PRXC3*, *PER42*, and *PER69* were highly expressed at 12 h. *PER56*, *PER45*, and *PER72* were highly expressed at 0 h; *PER49*, *PER52*, and *PER21* were all down-regulated; *PER32* expression was the highest at 32 h; *CAT2* and *CAT3* were up-regulated in the CATs gene family; APXs gene family members *APX1.1*, *APX1.2*, *APX3*, and *APXS* were expressed at 12 h. When treated with MT for 6 h, *APX1.1*, *APX1.2*, and *APX3* had higher expression levels than 0 h, and these genes are also involved in encoding peroxisomes. Compared with 0 h, the gene *MDAR1* of dehydroascorbate reductase (Monodehydroascorbate reductase, MDAR) had a higher expression level at 20 h and the lowest expression level at 12 h.

### Validation of transcriptome data by RT-qPCR analyses

**Fig. 7:**
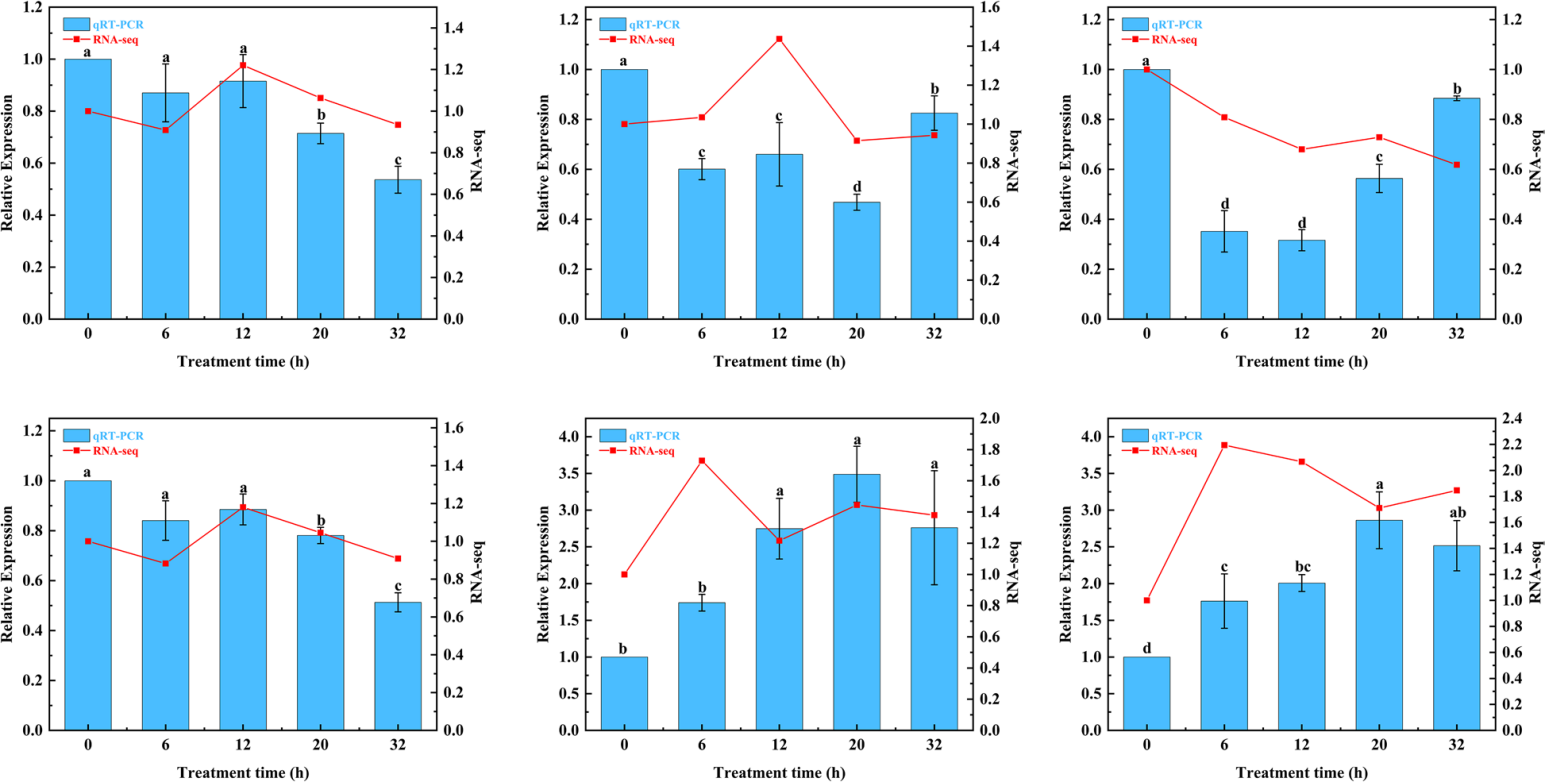
Expression of NO and H_2_O_2_ synthesis-related genes by qRT-PCR. Different lowercase letters indicated significant differences (*P* ≤ 0.05)

We verified the expression of NO and H_2_O_2_ synthesis-related genes by qRT-PCR, including *NOA1*, *BoCuAO1*, *NIA1*, *POX32*, *CAT3.1*, and *CAT3.2* (Fig. 7). The qRT-PCR results matched well with the transcriptome data.

### Effects of NO synthase inhibitors on NO, GRA, and SF synthesis in hairy roots of broccoli under MT treatment

#### Effects of NO synthase inhibitors on NO synthesis in hairy roots of broccoli under MT treatment

**Fig. 8:**
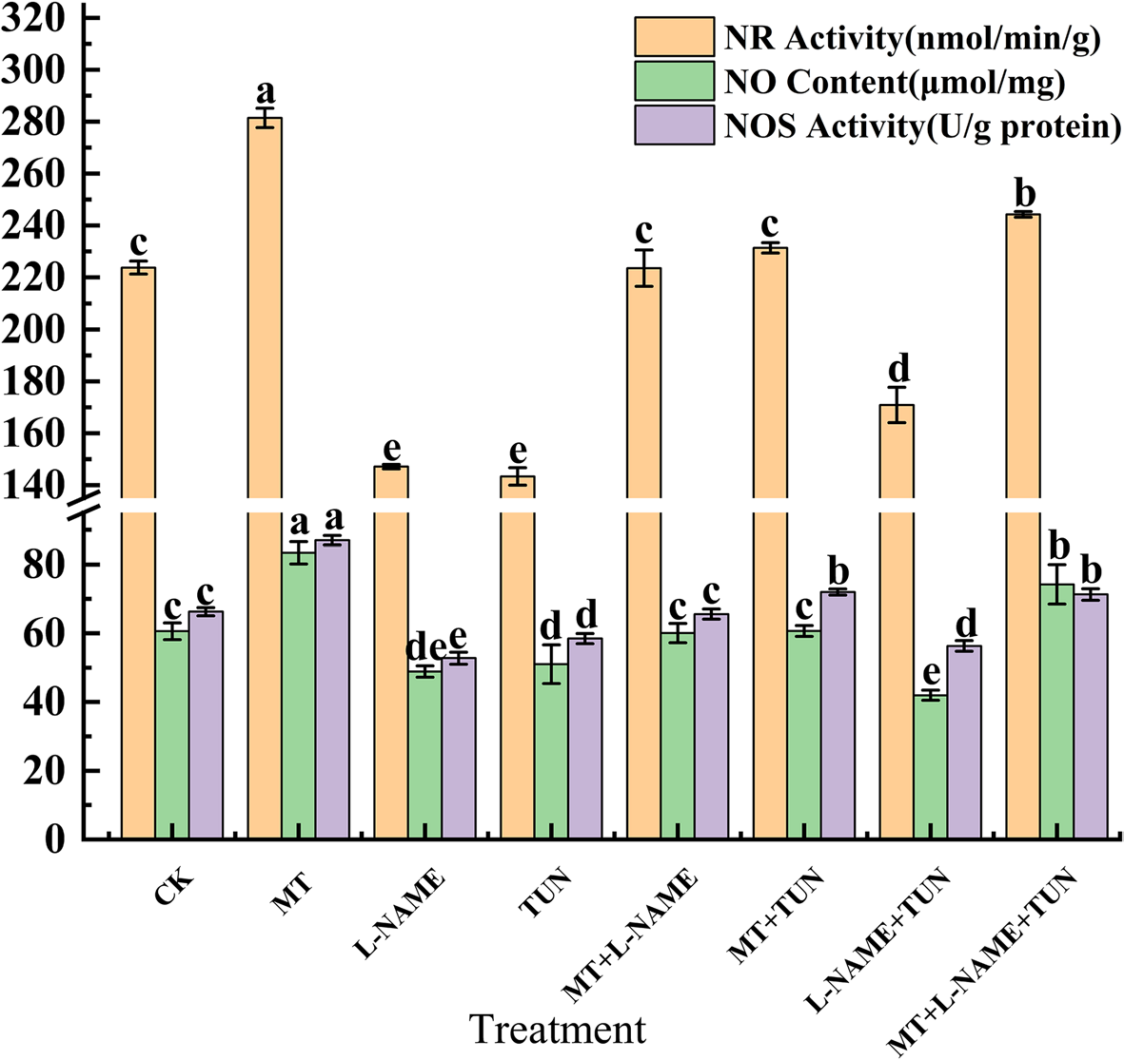
The effects of different enzyme inhibitors on NO synthesis induced by MT

The effect of NO synthase inhibitor on NO synthesis in hairy roots of broccoli under MT treatment is shown in Fig. 8. Compared with the control group, MT alone treatment significantly increased the activity of NOS, NR, and NO content (*P* ≤ 0.05). The NOS and NR activities of the L-NAME, TUN, and L-NAME + TUN treatment groups all decreased to varying degrees, indicating that L-NAME and TUN can effectively inhibit the production of NO in hairy roots of broccoli. Compared with L-NAME and TUN, the activity of NOS and NR increased in the MT + L-NAME + TUN-treated group, and the NO content also increased, indicating that MT inhibited L-NAME and TUN on NO synthesis in tissue cells The action has a mitigating effect.

### Effects of NO synthase inhibitors on GRA and SF production in hairy roots of broccoli under MT treatment

**Fig. 9:**
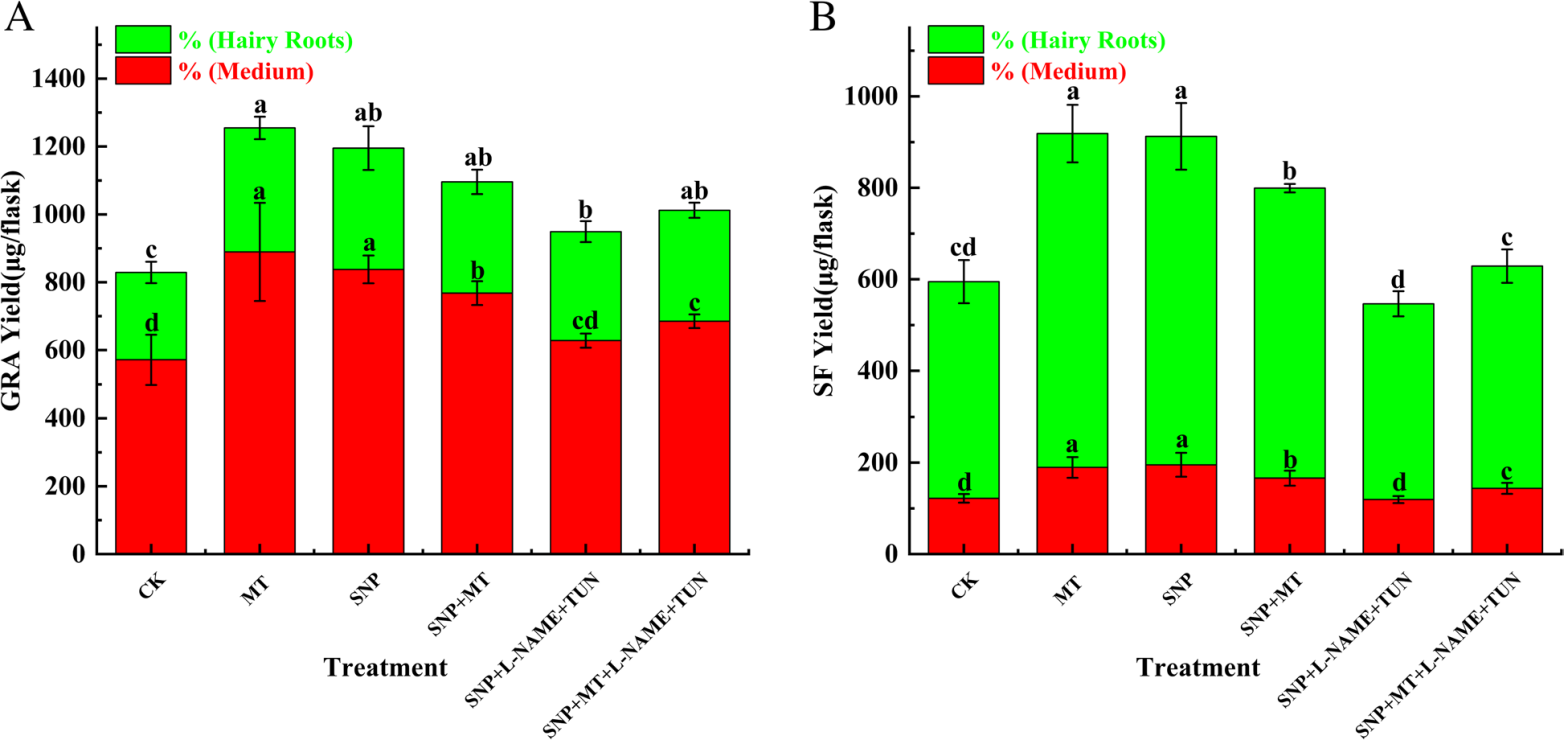
Effect of NO enzyme inhibitor on GRA and SF yield of broccoli hairy roots under MT treatment. (**A**) The yield of GRA. (**B**) The yield of SF

The effects of NO synthase inhibitors on the accumulation of GRA and SF in hairy roots of broccoli under MT-induced treatment are shown in Fig. 9. Compared with the control group (CK), MT alone significantly promoted the synthesis of GRA (*P* ≤ 0.05), while the total production of GRA in the NO synthase inhibitor treatment groups (L-NAME, TUN, and L-NAME + TUN) decreased by 11.94%, 10.24% and 10.98%, respectively, compared with the control group. The total yield of GRA in the + TUN treatment group, MT combined with L-NAME, and TUN treatment groups increased. The total SF yield of hairy roots of broccoli treated with MT alone reached 987.79 μg/flask, an increase of 54.41% compared with the control group. The SF yields of the TUN and L-NAME + TUN treatment groups were lower than that of the control group, and the SF yield of MT combined with the L-NAME and TUN treatment group increased by 34.61% compared with the L-NAME + TUN treatment group. Therefore, according to the preliminary analysis of the results, we conclude that NO synthase inhibitors can affect the synthesis of GRA and SF in the hairy roots of broccoli.

## Discussion

**Fig. 10:**
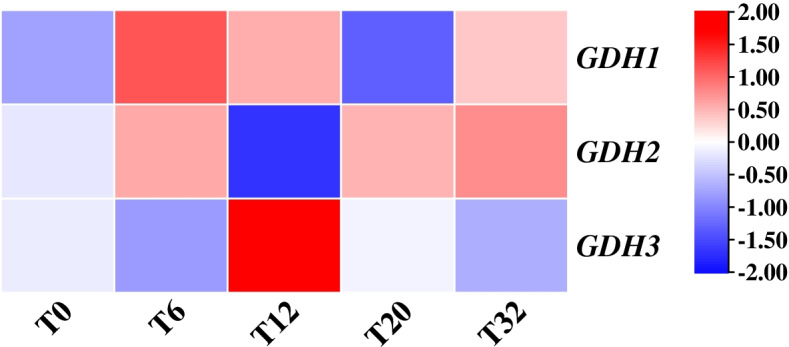
Heat map of nitrogen metabolism genes in broccoli hairy roots

NO is a signal molecule in plant cells. He and He [29] reported that NO and MT could interact with each other and participate in the plant growth process. In this study, we analyzed the expression of DEGs at the transcriptional level of broccoli hairy roots treated with exogenous MT for 0, 6, 12, 20, and 32 h. The GO annotations indicated that the DEGs involved in MT treatment of hairy roots of broccoli were mainly related to catalytic activity, cell, and metabolic process, and fourth-site enrichment using the exogenous signal substance MT to treat broccoli hairy roots will cause changes in the content of NO in hairy roots, which may further affect the synthesis of GRA and SF in hairy roots. In addition, our KEGG pathway enrichment results indicated that MT affected plant amino acid metabolism (Fig. 4). Wan et al. [30] found that MT promoted primary nitrogen assimilation by up-regulating the expression of two glutamate dehydrogenase (GDH) synthesis genes, *GDH1* and *GDH2*, in the primary nitrogen metabolism pathway in the transcriptome data of *Arabidopsis thaliana* processed by MT. In this experiment, *GDH1*, *GDH2*, and *GDH3* genes were also identified in the transcriptome data of broccoli hairy roots treated with MT. Among them, *GDH1* and *GDH2* were up-regulated at 6 h, and *GDH3* was up-regulated at 12 h after MT treatment (Fig. 10), indicating that exogenous application of MT to broccoli hairy roots promoted plant nitrogen metabolic processes.

Moreau et al. [31] showed that the *NOA1* gene was involved in synthesizing AtNOS1 protein and detected reduced NO content in *Arabidopsis NOA1* loss-of-function mutants. The results of the transcriptome data analysis in this study showed that during MT treatment of broccoli hairy roots, the expression trend of NOS encoding gene *NOA1* was first down-regulated, then up-regulated, and then down-regulated, and the expression level was the highest when MT was treated for 12 h, indicating that MT was up-regulated the expression of NOS encoding gene *NOA1* increases the NO content (Figs. 4A, 5, and 6).

The *NIA* gene family encodes NR synthesis genes. The data of Erdal. [32] showed that the activity of NR and NiR increased after MT treatment of maize seedlings, and the expression of the encoding genes *NR* and *NiR* were up-regulated. In this study, the NR gene family members *NIA2.1* and *NIA2.2* showed similar expression trends, both of which first decreased, then increased, and then decreased. The expression of *NIA1* was up-regulated at 20 h, while the expression of *NIA1* was generally down-regulated at other time points (Fig. 4B). The expression patterns of *NIA1* and *NIA2* were different, which is similar to the results of Olas and Wahl. [33]. The expression patterns of *NIA1* and *NIA2* in the apical meristem of *Arabidopsis* barely overlapped; therefore, it was speculated that the expression trends of *NIA1* and *NIA2* were different. This is because *NIA1* and *NIA2* respond differently to MT; Nitrite reductase is encoded by the *NIR* gene. In this study, *NIR1.1*, *NIR1.2*, and *NIR1.4* showed similar expression trends at the transcriptional level. The highest expression level was observed at 12 h MT treatment compared to 0 h, followed by a down-regulation of the expression level. There was a difference in the expression of *NIR1.3*, *NIR1.5*, *NIR1.6*, and *NIR1.7*, indicating that the expression of NiR gene family members was specific. Another study reported that nitrite-NO reductase (NiNOR) activity was found in the plasma membrane of tobacco roots [34]. This is different from the results of this study, and it may be due to species differences. *BoCuAO1* is a gene for the synthesis of copperamine oxidase. Copperamine oxidase is an important enzyme that catalyzes the degradation of polyamines. It plays an important role in the response of plants to external stimuli [35]. In this experiment, the expression of the *BoCuAO1* gene was up-regulated in MT-treated broccoli at 12 h in hairy roots (Fig. 4A), indicating that exogenous addition of MT may trigger plant resistance to stress.

**Fig. 11:**
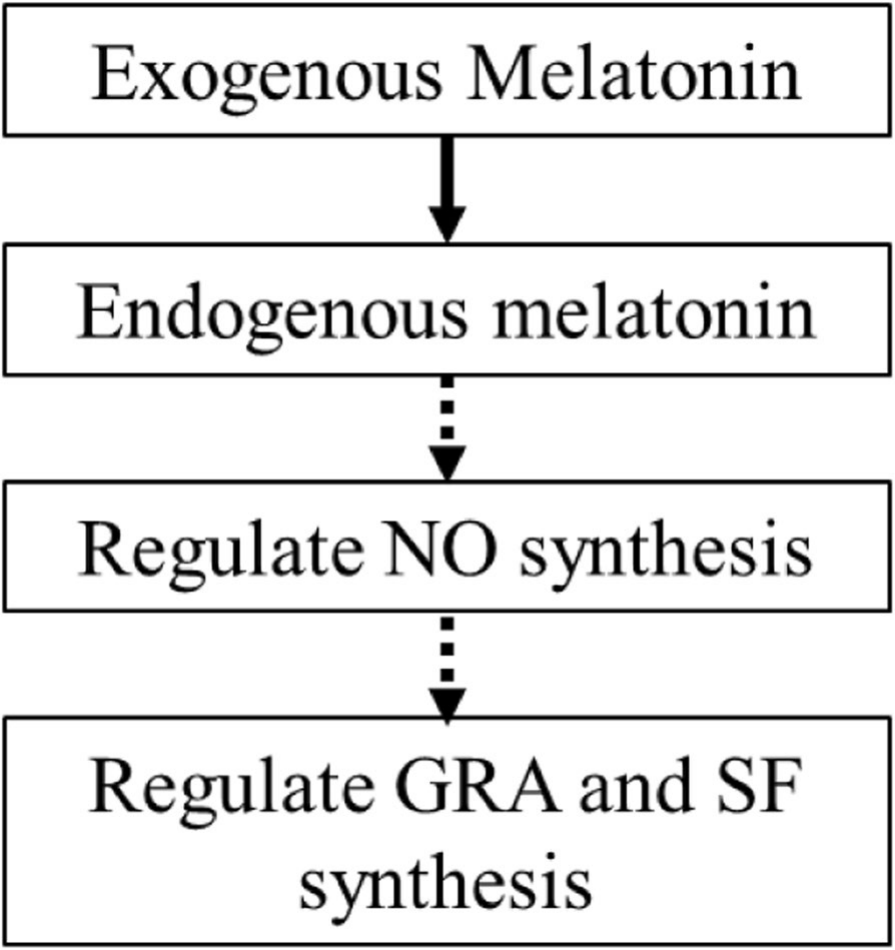
The simulated module of MT regulating the GRA and SF biosynthesis by NO in hairy roots of broccoli

Khan et al. [36] reported that MT controlled the level of ROS by regulating the activity of oxidoreductase. The synthesis of CAT encoding CAT was detected at the transcriptome level of broccoli hairy roots; two coding genes were identified in broccoli hairy roots under MT treatment: *CAT2* and *CAT3*. Compared with other treatments, the highest expression level of *CAT2* was at 12 h, and the highest expression of *CAT3* was at 6 h. The number of *PRXs* gene family members identified after MT treatment was high and differentially expressed. In addition, three *APXs* gene family members were identified based on transcriptome data analysis. Compared with 0 h, *APX1.1*, *APX1.2*, and *APX3* expressions were higher at 6 h after MT treatment, while *APXS* had the highest expression level at 0 h (Fig. 4B), indicating that the addition of MT treatment induces the expression of these three gene family members encoding different antioxidant enzymes. The specific gene expression indicated that MT treatment might induce the production of ROS in broccoli hairy root cells. Therefore, it is speculated that H_2_O_2_ may be involved in synthesizing GRA and SF under MT treatment (Fig. 11). In the experiment, the NO synthase inhibitors L-NAME and TUN effectively inhibited the enzymatic activities of NOS and NR and reduced the NO levels, while the production of GRA and SF also decreased, which is consistent with the study of NO synthesis [37]. The enzyme inhibitor treatment reduced the NO content and the content of secondary metabolite sulforaphane in broccoli, similar to the results of other studies. Therefore, it is speculated that NO synthase inhibitors affect the NO level in plant cells by inhibiting the activities of NOS and NR, thereby affecting the synthesis of GRA and SF. In addition, although the NO content in hairy roots is decreased, NO still exists in plant tissues, indicating that the NOS pathway and NR pathway are the main synthesis pathways of NO, but that there are other NO synthesis pathways. The NO content of the hairy roots treated with MT and NO synthase inhibitor in the experiment increased compared to the NO synthase inhibitor-treated group, indicating that MT can alleviate the decrease in NO concentration caused by NO synthase inhibitor and promote NO synthesis.

## Conclusion

In summary, the GO annotations associated with the 500 μmol/L MT treatment indicated that the DEGs involved in MT treatment of broccoli hairy roots were mainly related to catalytic activity, cell, and metabolic process. The KEGG pathway analysis indicated that MT treatment might affect the hormone signal transduction process in broccoli hairy roots. Broccoli hairy roots were treated with 500 μmol/L MT for 0, 6, 12, 20, and 32 h, respectively, and compared with 0 h, the production of GRA and SF increased under all other treatments. The highest yields of GRA and SF occurred at 12 h. The NO content was the highest at 12 h, and the H_2_O_2_ content was positively correlated with the MT concentration; when broccoli hairy roots are treated with melatonin, it induces the production of endogenous NO, which promotes the biosynthesis of glucoraphanin and sulforaphane. NO synthase inhibitor (L-NAME and TUN) could effectively inhibit the content of NO in broccoli hairy roots and reduce GRA and SF yield; MT could regulate NO levels by regulating NO synthesis-related enzymes and could alleviate the reduction of NO content in tissue cells caused by NO synthase inhibitor and promote NO synthesis.

## Methods

### Plant materials and treatment

The broccoli leaves were obtained from the sterile seedling line preserved at our laboratory at Gansu Agricultural University in Lan Zhou, Gansu Province, China. The establishment of broccoli hairy roots was derived from aseptic leaves of broccoli infected with *Agrobacterium rhizogenes* (ATCC15834), as previously reported [38]. Samples of the hairy roots were cultured in 150 mL beaker flasks containing 100 mL of MS liquid medium on an orbital shaker at 110 rpm/min in the dark at 25 °C.

For MT treatment, MT (Sigma, CA, USA, CAS: 73–31-4) stock solution was filter-sterilized through 0.22-μm filters and added to cultures of 20-days-old hairy roots to a desired final concentration of 500 μmol/L. Hairy roots were collected after 0, 6, 12, 20, and 32 h of MT treatment for qRT-PCR, HPLC analysis, and transcriptome analysis. All treatments were performed in three independent biological replicates.

The NOS inhibitor L-NAME (Yuanye, Shanghai, China, CAS: 51298–62-5) and NR inhibitor TUN (Yuanye, Shanghai, China, CAS: 7790–60-5) were dissolved in sterile water in an ultra-clean bench and filtered through a 0.22-μm microporous filter membrane, respectively. L-NAME and TUN were added to the liquid medium according to their concentration of 1 μmol/L in the liquid medium. The addition time was 30 min before MT induction treatment, and each group of experiments was repeated three times.

To verify the effect of NO, the NOS inhibitor L-NAME, NR inhibitor TUN, and SNP were added when the hairy roots were grown for 20 days (growth resting period). The materials were collected after 12 h of treatment for subsequent experiments. The specific treatment combinations are shown in Table 1.

**Table 1:** Experimental treatment

Treatment	MT(μmol/L)	L-NAME(μmol/L)	TUN(μmol/L)	
1	-	-	-	
2	500	-	-	
3	-	1	-	
4	-	-	1	
5	500	-	1	
6	-	1	1	-
7	500	1	-	-
8	500	1	1	-

### HPLC Analysis of glucoraphanin and sulforaphane

We followed the method described in Tian et al. [39] to extract and detect glucoraphanin and sulforaphane in hairy roots.

#### RNA-seq library construction and sequencing

Hairy roots were treated by MT for 0, 6, 12, 20, and 32 h. The MT-treated hairy roots and control samples (0 h) were collected from three biological replicates and analyzed by transcriptomic-based technology. RNA concentration and purity were measured using a NanoDrop 2000 device (Thermo Fisher Scientific, Wilmington, DE). RNA integrity was assessed using the RNA Nano 6000 Assay Kit of the Agilent Bioanalyzer 2100 system (Agilent Technologies, CA, USA).

### Identification of differentially expressed genes (DEGs) and functional enrichment

The reference genome and gene model annotation files of broccoli (*Brassica oleracea* L. var. *botrytis* L Planch) were downloaded from genome websites (GCA_900416815.2). Fragments per kilobase of transcript per million mapped reads (FPKM) were used to determine the relative expression levels of each gene. Differential expression analyses of two conditions/groups were performed using DESeq2-EBSeq. DESeq2-EBSeq provides statistical routines for determining differential expression in digital gene expression data using a model based on the negative binomial distribution. The resulting p-values were adjusted using Benjamini and Hochberg’s approach for controlling the false discovery rate. Genes with an adjusted FDR = 0.05 found by DESeq2-EBSeq were assigned as differentially expressed. The expression levels of DEGs were considered significantly differentially expressed genes with an adjusted FDR = 0.05 and |foldchange|= 1.5. The GO term enrichment of DEGs was evaluated using the GOseq R package [40]. Statistical enrichment of DEGs in the KEGG[41] was identified with KOBAS [42]. Then Mapman program was used to analyze the transcriptome data of metabolic and signal pathways.

### qRT-PCR validation

Six DEGs specific primers were designed by primer 5 (Table 2). Total RNA was isolated using an RNA kit (Tiangen DP432). cDNA synthesis and qRT-PCR analysis were performed using a one-step SYBR Prime script plus an RT-PCR kit (Tiangen FP209). According to the following scheme, PCR amplification was performed in a 96-well platform (Roche LC 96 in Switzerland): 180 s at 95 °C, 5 s at 95 °C, 15 s at 60 °C, and 40 cycles. The melting curve analysis was performed at 60 ~ 95 °C. Total RNA concentrations and gel electrophoresis were used to reverse transcription. According to the Ct value of the target gene and the internal reference gene, the gene expression level was calculated by the 2^−ΔΔCt^ method [43]. The *ADF3* gene was used as the endogenous control [44]. All assays for each gene were performed in triplicate under identical conditions.

**Table 2:** Primer sequences used in the qRT-PCR analysis

Gene name	Primer sequences (5' to 3')	Accession
*ADF3*	Forward: GAGGAGCAGCAGAAGCAAGTGG Reverse: ATCGGCATTCATCAGCAGGAAGAC	XM_013797493.2
*POX32*	Forward: TCAGTCGAGTTGGCAGGAGGTC Reverse: AAGCGTGAAGAATGGAGCAGGAAG	XM_013750548.1
*CAT3.1*	Forward: CCACGCCTTGAAACCGAACCC Reverse: CATCCATGTGCCTGTAGTCCTGTG	XM_022705954.1
*CAT3.2*	Forward: CCATGAGGGATATTCGTGGCTTCG Reverse: GTCTTCGGGTTCGGCTTCAGTG	XM_013871970.2
*NOA1*	Forward: TTACCCGCCCTTGCTCCTCAG Reverse: TCCTGACGAGACCTCCCCAAAAG	XM_013876162.2
*NIA1*	Forward: ACGAGGACGAGAGCCACAACC Reverse: AGACGCACCATAGAGGAGTTACGG	XM_013794573.2
*BoCuAO1*	Forward: TTGAACGATACGCAGGCGATGTC Reverse: GTAGCCATCCGAGCGACCAATG	XM_013773883.1

### NO content determination

NO content was determined using a NO content determination kit (Suzhou Keming Biological Co., Ltd., product number: NO-2-G), and 100 mg of hairy root material was accurately weighed. We then added 1 mL of pre-cooled sodium phosphate (50 mmol/L, pH 7.0) buffer to grind the homogenate in an ice bath and centrifuged this at 10,000 rpm at 4 °C for 15 min. The supernatant was taken for NO determination.

### Determination of NOS viability

NOS viability was determined using a NOS viability assay kit (Nanjing Jiancheng, item no. A014-2). Two hundred milligrams of material were homogenized by grinding in 2 mL of pre-chilled PBS (50 mmol/L, pH 7.0) buffer on ice and then centrifuged at 12,000 rpm for 15 min at 4 °C. The supernatant was taken for NOS viability determination.

### NR viability assay

The NR viability assay kit (Suzhou Kemin Biological Co., Ltd., item no. NR-2-Y) was used for NR viability determination. One hundred milligrams of material was homogenized by grinding in 1 mL of pre-chilled PBS (50 mmol/L, pH 7.0) buffer in an ice bath and centrifuged at 8000 rpm for 10 min at 4℃. The supernatant was taken for NR viability determination.

### H_2_O_2_ content determination

The H_2_O_2_ content was determined using a commercial kit (Suzhou Keming Biological Co., Ltd., product number: H_2_O_2_-2-Y). One hundred milligrams of the material were accurately weighed. The homogenate was ground with 1 mL of pre-cooled sodium phosphate (50 mmol/L, pH 7.0) buffer in an ice bath and centrifuged at 8000 rpm at 4 °C for 10 min. The supernatant was taken for H_2_O_2_ determination.

### Data analysis

The experimental data were analyzed using SPSS 21.0 software, and statistically significant differences were identified using Duncan’s new complex range method (*P* ≤ 0.05). The data were processed, and figures and tables were made using Microsoft Excel 2016 and Origin 2019. Photos were edited using Photoshop 2018.

## Supplementary Information

**Additional file 1.** Table S1. Sequencing data and quality statistics of 15 transcriptomes at five time points of MT-induced hairy roots of broccoli.

## Data Availability

The raw sequencing data has been uploaded to the Sequence Read Archive (https://www.ncbi.nlm.nih.gov/sra) under Bioproject PRJNA796369 and PRJNA764437. The reference genome and gene model annotation files of Broccoli (*Brassica oleracea* L. var. *botrytis* L Planch) were downloaded from genome websites (GCA_900416815.2).

## Abbreviations

GLS: Glucosinolates
GRA: Glucoraphanin
SF: Sulforaphane
MT: Melatonin
ITC: Isothiocyanates
DEGs: Differentially expressed genes
GO: Gene ontology
KEGG: Kyoto Encyclopedia of Genes and Genomes
NCBI: National Center for Biotechnology Information
qRT-PCR: Quantitative real-time polymerase chain reaction
NO: Nitric oxide
NOS: Nitric oxide synthase
NR: Nitrate reductase
NiR: Nitrite reductase
ROS: Reactive oxygen species
IAA: Indole-3-acetic acid
SOD: Superoxide dismutase
CAT: Catalase
APX: Ascorbateperoxidase
TUN: Potassium tungstate
L-NAME: N-nitro-L-arginine methyl
