# Supplementary material for: Glucoraphanin and sulforaphane biosynthesis by melatonin mediating nitric oxide in hairy roots of broccoli (Brassica oleracea L. var. italica Planch): insights from transcriptome data
Source: BMC Plant Biol. 2022 Aug 17;22:403. doi: 10.1186/s12870-022-03747-x (PMC9382772; doi:10.1186/s12870-022-03747-x)
Supplement: Supplementary file 1 — Additional file 1. Table S1. Sequencing data and quality statistics of 15 transcriptomes at five time points of MT-induced hairy roots of broccoli. [file 12870_2022_3747_MOESM1_ESM.docx]

Table. S1 Sequencing data and quality statistics of 15 transcriptome at 5 time points of MT induced hairy roots of Broccoli. Samples: sample name; clean reads: the total number of pair end reads in clean data; clean bases: the total base number of clean data; Q20: the sequencing error rate of bases is 1 /100; Q30: the sequencing error rate of bases is 1 / 1000; GC content: the proportion of guanine and cytosine in the four bases of DNA.

| Samples | Clean reads(10^6^) | Clean bases(10^9^) | GC Content(%) | Q20(%) | Q30(%) |
| --- | --- | --- | --- | --- | --- |
| T0a | 30.55 | 8.70 | 47.24 | 97.88 | 94.37 |
| T0b | 24.29 | 6.92 | 47.38 | 97.86 | 94.32 |
| T0c | 25.66 | 7.31 | 47.22 | 97.88 | 94.32 |
| T6a | 23.18 | 6.61 | 47.00 | 97.86 | 94.27 |
| T6b | 22.41 | 6.38 | 47.13 | 98.01 | 94.56 |
| T6c | 25.35 | 7.20 | 47.17 | 97.80 | 94.18 |
| T12a | 24.51 | 6.98 | 47.00 | 97.99 | 94.46 |
| T12b | 29.22 | 8.31 | 47.08 | 97.95 | 94.39 |
| T12c | 24.11 | 6.86 | 47.25 | 97.93 | 94.42 |
| T20a | 24.13 | 6.87 | 47.15 | 97.85 | 94.25 |
| T20b | 23.58 | 6.72 | 47.05 | 97.84 | 94.15 |
| T20c | 21.75 | 6.20 | 47.00 | 97.84 | 94.15 |
| T32a | 21.35 | 6.07 | 47.03 | 97.84 | 94.15 |
| T32b | 23.68 | 6.75 | 46.87 | 97.70 | 93.94 |
| T32c | 26.94 | 7.66 | 47.15 | 97.79 | 94.06 |
